# Agammaglobulinaemia despite terminal B-cell differentiation in a patient with a novel *LRBA* mutation

**DOI:** 10.1038/cti.2017.20

**Published:** 2017-05-26

**Authors:** Nashat Al Sukaiti, Khwater AbdelRahman, Jalila AlShekaili, Sumaya Al Oraimi, Aisha Al Sinani, Nasser Al Rahbi, Vicky Cho, Matt Field, Matthew C Cook

**Affiliations:** 1 Department of Pediatrics, Allergy and Clinical Immunology Unit, Royal Hospital, Muscat, Oman; 2 Department of Pediatrics, Allergy and Clinical Immunology, Royal Hospital, Muscat, Oman; 3 Department of Microbiology and Immunology, Sultan Qaboos University Hospital, Seeb, Oman; 4 Department of Pediatrics, Respirology Unit, Royal Hospital, Muscat, Oman; 5 Department of Pediatrics, Endocrine Unit, Royal Hospital, Muscat, Oman; 6 Department of Pathology, Royal Hospital, Muscat, Oman; 7 Department of Immunology, The John Curtin School of Medical Research, Australian National University, Acton, Australian Capital Territory, Australia; 8 Department of Immunology, Canberra Hospital, Canberra, New South Wales, Australia

## Abstract

Mutations in lipopolysaccharide-responsive vesicle trafficking, beach and anchor-containing protein (LRBA) cause immune deficiency and inflammation. Here, we are reporting a novel homozygous mutation in *LRBA* allele in 7-year-old Omani boy, born to consanguineous parents. He presented with type 1 diabetes, autoimmune haematological cytopenia, recurrent chest infections and lymphocytic interstitial lung disease. The patient was treated with CTLA4-Ig (abatacept) with good outcome every 2 weeks for a period of 3 months. He developed complete IgG deficiency, but remarkably, histological examination revealed germinal centres and plasma cells in lymphoid and inflamed lung tissue. Further charatecterisation showed these cells to express IgM but not IgG. This *ex vivo* analysis suggests that *LRBA* mutation confers a defect in class switching despite plasma cell formation.

Lipopolysaccharide-responsive vesicle trafficking, beach and anchor-containing protein (LRBA) is one of nine known mammalian BEACH domain-containing proteins (BDCP). BDCPs serve as scaffolds for multi-molecular complexes that are important for guiding and regulating vesicular trafficking.^1, 2^ So far, mutations in the genes encoding four BDCP family members have been reported to cause Mendelian disease (*LYST*, *NBEAL2*, *WDR81* and *LRBA*). Homozygous or biallelic mutations in *LRBA* have been described in patients with early onset and often severe autoimmunity and antibody deficiency.^3^ LRBA regulates intracellular trafficking of CTLA, a negative regulator of T-cell immunity.^4^ Reported cases arising from *LRBA* mutations exhibit considerable phenotypic variation, even in individuals carrying the same mutation, and histopathological abnormalities include granulomas in skin, lungs and central nervous system, and lymphocytic infiltrates of lung and lamina propria in the gut.^5, 6, 7, 8, 9^

Recurrent infections occur in the majority, and immune-mediated thrombocytopenia, enteropathy and hypogammaglobulinaemia, each reported in ~50% of cases.

Reported immunologic findings are variable, and include decreased B cells, and deficiency of CD4^+^ T-regulatory (Treg) cells.^10^ Here, we report a novel homozygous nonsense mutation of *LRBA* resulting in autoimmunity and immune deficiency. We report the presence of normal germinal centre formation and, more remarkably, plasma cells in lymph node and inflamed parenchymal tissue despite agammaglobulinaemia.

## Case report

**Figure 1:**
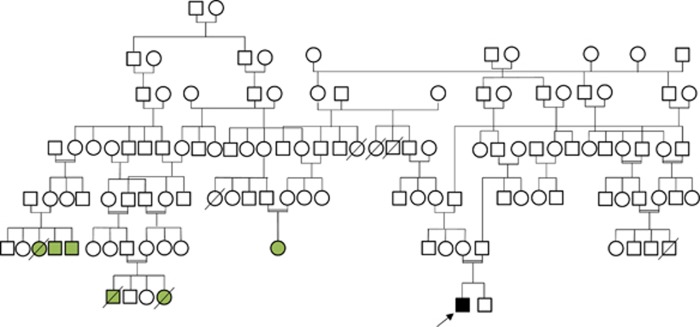
Family pedigree of the proband (black and arrow) and the relatives with MHC class II deficiency (green).

A 7-year-old Omani boy born to a consanguineous parent presented at the age of 9 months with type 1 diabetes, and was started on insulin replacement therapy. At the same time, he was found to have hepatosplenomegaly. He had two younger healthy male siblings. There was a family history of major histocompatibility complex class II deficiency due to a *CIITA* missense mutation (M1071T); neither proband nor his parents carried this mutation (Figure 1). At the age of 2 years, he developed repeated episodes of immune-mediated thrombocytopenia, which were managed successfully with high-dose intravenous immunoglobulin and prednisolone (Table 1). He remained in remission from immune-mediated thrombocytopenia for 2 months then developed Evan’s syndrome, (direct Coomb’s test-positive autoimmune haemolytic anaemia and immune-mediated thrombocytopenia), which failed to respond to either intravenous immunoglobulin or pulse methylprednisolone. At the age of 3 years, he was treated with four doses of rituximab, which induced a sustained remission. At the age of 4 years, a year after the last dose of rituximab treatment, however, he developed bacterial infections, which became recurrent. These included tonsillitis, otitis media, three bouts of pneumonia and one episode of acute salmonella gastroenteritis. At the age of 5 years, he was admitted with tachypnea and shortness of breath.

**Figure 2:**
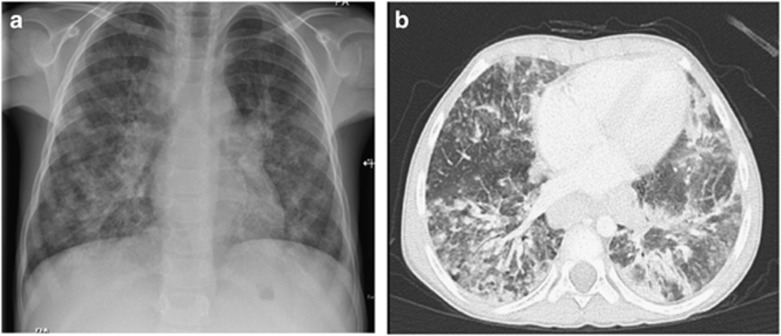
Radiological findings. Chest X-ray and high-resolution computed tomography scan showing interstitial lung infiltrates and hilar lymphadenopathy.

Chest X-ray at the age of 5 years showed interstitial lung infiltrates and hilar lymphadenopathy (Figure 2a). Bronchoalveolar lavage was positive for cytomegalovirus by PCR, however, there were no inclusion bodies seen by histopathology. At that time, cytomegalovirus DNA was detected at only 75 copies per ml in serum by PCR, however, this increased to 2434 copies per ml 1 month later. He was treated with intravenous ganciclovir 5 mg kg^−1^ twice a day followed by oral valganciclovir 15 mg kg^−1^ twice a day for a total of 6 weeks. Cytomegalovirus DNA was undetectable after treatment. *Aspergillus niger* was isolated from bronchoalveolar lavage. Blood cultures were negative. He received 6 weeks treatment with voriconazole after which repeated bronchoalveolar lavage was negative for aspergillus. Despite administration of appropriate antimicrobial therapy, radiological changes persisted. High-resolution chest computed tomography scan was consistent with lymphocytic interstitial pneumonitis (Figure 2b).

**Figure 3:**
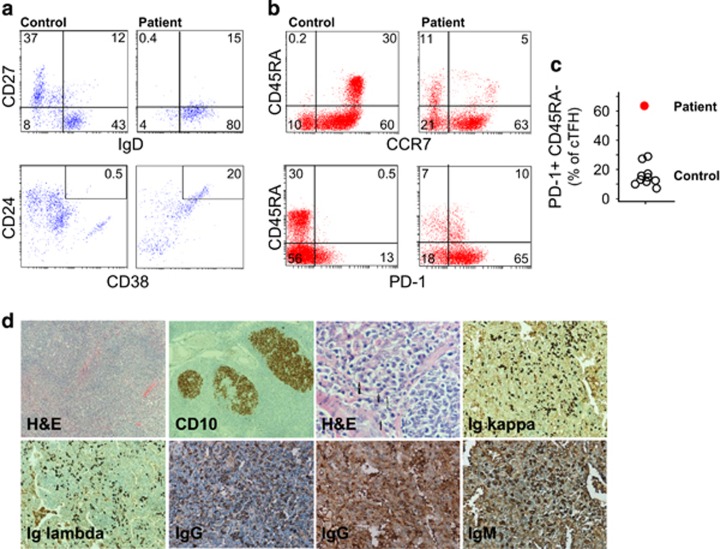
T- and B-cell phenotypes and tissue plasma cell infiltrate. (**a**) Flow cytometry analysis of B-cell subsets; naive (CD19^+^CD27^−^) and memory (CD19^+^CD27^+^) B cells, CD21^low^ CD19^+^ B cells in peripheral blood mononuclear cells (PBMCs) from patient and a healthy control. (**b**) CD4^+^ T-cell subsets (naive, CCR7^+^CD45RA^+^; T_CM_, CCR7^+^CD45RA^−^; T_EM_, CCR7^−^CD45RA^−^; T_EMRA_, CCR7^−^CD45RA^+^) and cTFH (CD45RA^−^PD-1^+^) from patient and a healthy control. (**c**) Summary for the cTFH obtained for the proband and normal controls (*n*=10). (**d**) Biopsies from lymph node with follicular hyperplasia showing reactive secondary follicles (H&E, 50 ×). Lymph node immunohistochemistry showing established germinal centres, CD10 immunoperoxidase (50 ×), and kappa and lambda light chain and immunoglobulin isotype expression.

Investigations at age 2 years (before rituximab treatment) revealed marked polyclonal hypergammaglobulinaemia (IgG >32.2 g l^−1^), with normal IgA, IgM and IgE (Table 1). At age 5 years (2 years after rituximab treatment), he was noted to be agammaglobulinemic. Lymphocyte subset analysis performed at age 5 years showed absence of CD20^+^ B cells, which started to recover by the age of 6 years old. Flow cytometric assessment of peripheral B-cell subsets at the age of 7 years revealed reduction in memory B cells and increased CD21^+^ B cells compared to normal controls of similar age group (Figure 3a). Transitional B cells were evident (CD24^hi^ CD38^hi^). Flow cytometric analysis of different T-cell subsets revealed marked reduction of naive cells in both CD4^+^ and CD8^+^ T-cell compartments, an inverted ratio of CD4:CD8T cells (Table 2). Moreover, there was an expansion of circulating follicular helper T cells (cTfH) marked by high number of CD4^+^ PD-1^+^CD45RA^−^ compared to normal controls (*n*=10) (Figures 3b and c).

Biopsies from lung obtained when the patient was 5 years old revealed type two pneumocyte hyperplasia. Alveolar septa were markedly expanded, with nodular lymphoid aggregates containing germinal centres identified by H&E. A cervical lymph node biopsy obtained at the same time revealed follicular hyperplasia with histiocytic microgranulomas and aggregates of monocytoid B cells. The lymphoid follicles showed prominent germinal centres confirmed by immunohistochemistry (CD10^+^). Abundant Ig kappa and Ig lambda positive plasma cells were identified, consistent with a polyclonal plasma cell response, although no IgG^+^ cells were present (Figure 3d). Bone marrow examination was hypercellular with plasma cells present and no other specific changes.

**Figure 4:**
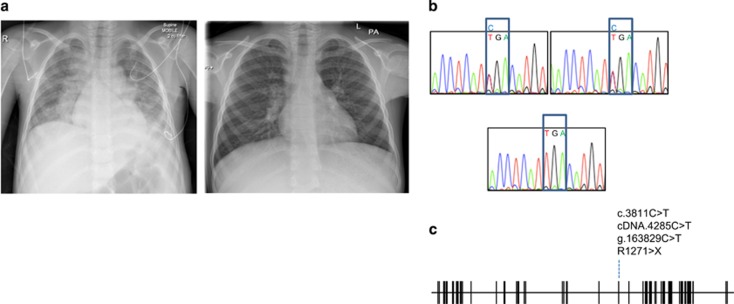
Diagnosis and therapy. (**a**) Comparison of chest X-rays taken before and 3 months after abatacept therapy. (**b**) DNA chromatograms of *LRBA* showing heterozygous mutations in the parents (upper panels) and homozygous mutation in the proband. (**c**) Location of nonsense mutation in exon diagram of *LRBA*.

Whole-exome sequencing was performed and analysed for known monogenic causes of primary immune deficiency or autoimmunity. We identified a novel homozygous nonsense mutation in *LRBA* (Chr4:R1271X; g.163829C>T; c.4285C>T). The mutation was confirmed by Sanger sequencing, and both parents were confirmed as heterozygous for the same mutation (Figures 4b and c). *LRBA*^R1271X^ has not been reported in 1000Genomes, dbSNP, ClinVar, ExAC or HGMD, and has not been detected in >200 in-house exome sequences from unrelated individuals. The stop is predicted to result in loss of protein due to nonsense mediated decay of LRBA transcripts. After obtaining this molecular diagnosis, the patient was treated with abatacept (10 mg kg^−1^) every 2 weeks for 3 months at the time of writing this article. He showed remarkable clinical improvement in term of his shortness of breath, and his weight gain as well as clearance of X-ray (Figure 4a).

## Discussion

We have reported a novel allele of *LRBA* presenting with type 1 diabetes, autoimmune cytopenias and severe lymphocytic pneumonitis. At initial assessment, the patient had polyclonal hypergammaglobulinaemia, but he subsequently became agammaglobulinaemic. Although he had been treated with rituximab, agammaglobulinaemia was noted >12 months after his treatment. He was commenced on intravenous immunoglobulin precluding further assessment of endogenous IgG production, but he remained IgA deficient for at least 4 years after rituximab. It is noteworthy that despite agammaglobulinaemia, histological examination of lung and lymph node revealed abundant germinal centres with plasma cells being present. While plasma cells appeared polyclonal by Ig kappa and Ig lambda staining, they were negative for IgG.

BDCPs are thought to assemble macromolecular complexes that regulate vesicular trafficking and function.^1^ The ultra structural signatures of *LRBA* mutation include disordered autophagy, resulting in increased apoptosis^3^ and altered trafficking of CTLA4.^4^ The significance of the latter observation is borne out by the efficacy of abatacept therapy, as illustrated by our case. Nevertheless, manifestations of LRBA deficiency vary substantially from patient to patient. In Chediak–Higashi syndrome, which results from mutations in the BDCP family member *LYST*, phenotypic variation has been related to genotype, with milder phenotypes in patients with missense mutations.^11^ While there is genetic heterogeneity among the *LRBA* mutant cases reported to date (~50), all reported cases arise from nonsense mutations, indels or large deletions.

Clinical resemblance to autoimmune lymphoproliferative syndrome, deficiency of Tregs and response to abatacept indicate that T-cell dysregulation is an important cause of autoimmunity and inflammation in LRBA deficiency. Furthermore, clinical improvement has been noted in at least two cases treated with hematopoietic stem cell transplantation, although it is interesting to note that recurrent or persistent thrombocytopenia and progressive vitiligo was reported even in the face of documented microchimerism.^12, 13^

Hypogammaglobulinaemia is common but not universal with LRBA deficiency. Indeed, as illustrated in our case, an increase in immunoglobulin can be observed early in the natural history. The pathophysiology of immune deficiency related to LRBA mutation remains incompletely understood. Remarkably, we have shown agammaglobulinaemia in the face of germinal centre formation, and present plasma cells. This suggests that within the B-cell compartment, immunoglobulin deficiency arises as a result of plasma cell dysfunction not deficiency. Future studies will be necessary to determine the nature of this functional defect.

## Methods

Approved hospital consent was taken for the genetic analysis for the patient and his parents.

Cellular T-and B-cell phenotype (flow cytometry): the following antibodies were used for flow cytometry staining from Beckman Coulter (Brea, CA, USA): anti-CD45-Krome Orange (clone J33), anti-CD19-Red-X (ECD) (clone J3-119), and anti-CD21-PE (clone BL13), anti-CD27-PC7 (clone 1A4CD27), and anti-CD38-APC-A750 (clone LS198-4-3) as Dura clone IM B cells. Anti-CD31-Pacific blue (clone 5.6E), anti-CD62L-APC750 (clone DREG56), anti-CD3-FITC (clone UCHT-1), CD4-PE (clone 13B8.2) and CD45-PC5.5 (clone J33). Anti-CD45-Krome Orange (clone J33), anti-CD3-APC-A750 (clone UCHT-1), antiCD4-APC (clone 13B8.2), antiCD8-A700 (clone B9.11), anti-CD45RA-FITC (clone 2H4), CCR7-PE (clone G043H7), anti-PD-1-PC5.5 (clone PD13.5), anti-CD57-PB (NC1) as Dura clone IM T-cell subsets from Beckman Coulter. Hundred microlitres of blood was added to the desired cocktail of antibodies and incubated for 20 min at room temperature. Hundred microlitres of lysing solution optilyse-B or VersaLyse was added according to the manufacture recommendation. This was followed up with a wash step and then acquisition of the sample using Navios flow cytometer (Brea, CA, USA).

### Whole-exome capture sequencing

The Illumina paired-end genomic DNA sample preparation kit (PE-102-1001, Illumina, San Diego, CA, USA) was used for preparing the libraries including end repair, A-tailing and ligation of the Illumina adaptors. Each sample was prepared with an index using the Illumina multiplexing sample preparation oligonucleotide kit (PE-400-1001, Illumina) and then pooled in batches of six in equimolar amounts prior to exome enrichment. The Illumina TruSeq exome kit (FC-121-1008, Illumina) was used to capture the human exome for each sample pool. Each 6-plex exome enriched library was sequenced in two lanes of an Illumina HiSeq 2000 with version 2 chemistry as 100 bp paired-end reads.

Sequence reads were mapped to the GRCh37 assembly of the reference human genome using the default parameters of the Burrows–Wheeler Aligner (bio-bwa.sourceforge.net).^14^ Untrimmed reads were aligned allowing a maximum of two sequence mismatches and reads with multiple mappings to the reference genome were discarded along with PCR duplicates. Sequence variants were identified with SAMtools (samtools.sourceforge.net)^15^ and annotated using Annovar (http://annovar.openbioinformatics.org).^16^

## Figures and Tables

**Table 1:** Summary of immunological laboratory findings

*Age in years*	*2 years*	*5 years*	*6 years*	*7 years*
*Test*				
IgG (3.5–12 g l^−1^)	>32.2	<1.0	4.5	6.9
IgA (0.15–1.6 g l^−1^)	1.7	<0.05	<0.05	<0.05
IgM (0.4–2 g l^−1^)	0.7	<0.05	1.65	4.99
IgE (0–90 IU ml^−1^)	<15			
Hep B antibody		NR		
Measles, mumps and rubella antibodies	NR	NR		
CD3 (1.0–2.0 × 10^9^)	ND	5.210	3.260	0.730
CD4 (0.5–1.3 × 10^9^)	ND	1.230	0.860	0.820
CD8 (0.3–1.0 × 10^9^)	ND	3.510	2.030	1.730
CD19 (0.2–0.5 × 10^9^)	ND	0.410	0.300	0.190
CD20 (6–23%)	ND	<1	2.7	3.6
CD16/56 (cells per μl)	ND	0.840	0.450	0.090
%CD3^+^CD4^+^				33.4% (27.7–46.3)
%CD4^+^CD45RA^+^CCR7^+^ (naive CD4 cells)				5.2% (29.2–66.5)
%CD4^+^CD45RA^−^CCR7^+^ (central memory CD4T cells)				63.3% (11.6–24.7)
%CD4^+^CD45RA^−^CCR7^−^ (effector memory CD4T cells)				20.7% (7.1–21.4)
%CD4^+^CD45RA^+^CCR7^−^ (terminally differentiated CD4T cells)				10.7% (6.9–34.4)
%CD3^+^CD8^+^				51.5% (15.7–33.8)
%CD8^+^CD45RA^+^CCR7^+^ (naive CD8T cells)				4.9% (8.6–50.1)
%CD8^+^CD45RA^−^CCR7^+^ (central memory CD8T cells)				4.5% (1–4.6)
%CD8^+^CD45RA^−^CCR7^−^ (effector memory CD8T cells)				44.4% (16–47.2)
%CD8^+^CD45RA^+^CCR7^−^				46.1% (19.5–52.5)
%CD3^+^CD4^+^CD62L^+^CD31^+^ (recent thymic emigrant)				9% (19.4–60.9)
%CD19^+^				7% (9.7–23.7)
% CD19^+^IgD^+^CD27^−^ (naive B cell)				80.1% (47.3–77.0)
%CD19^+^CD27^+^IgD^+^ memory B cell				15.4% (5.2–20.4)
%CD19^+^CD27^+^IgD^−^ memory B cell				0.4% (10.9–30.4)
%CD19^+^CD27^−^IgD^−^ memory B cell				4.0% (2.3–11.0)
%CD19^+^CD24^hi^CD38^hi^ transitional B cell				20.2% (4.6–8.3)
%CD19^+^CD21^lo^CD38^lo^				38% (2.3–10.0)
%CD19^+^CD24^lo^CD38^hi^ plasmablast				0.8% (0.6–5.3)

Abbreviations: ND, not done; NR, not reactive.

Reference ranges.^17, 18^

**Table 2:** Case histology chronology

*Age*	*Complication*	*Hb (g l^−1^)*	N *(× 10*^*9*^* l^−1^)*	*Plt (x10*^*9*^* l^−1^)*	*IgG (g l^−1^)*	*IgA (g l^−1^)*	*IgM (g l^−1^)*	*CD19 (× 10^9^ l^−1^)*	*Treatment*
0.9	T1D	13.3	1.8	250					Insulin
2.3	ITP	12.9	2.1	13	>30	1.7	0.7		IVIg
2.8	AIHA, ITP	7.0	1.6	1					IVIg, prednisolone
3.0	ITP	12.5	5.5	3					IVIg, prednisolone
3.2	ITP	13.6	3.9	21					RTX
4.2	Pneumonia, OM, gastroenteritis	12.9	3.2	255	<1	0.05	0.05	0	Antibiotics
5.2	Pneumonia (CMV), LIP, HS	11.1	2.1	148	4.5	0.05	1.65	0.3	GCV, VCZ, IVIg, MP
6.5	Pneumonia (*Haemophilus* *influenzae*), LIP, HS	11.8	2.4	171	6.9	0.05	4.99	3.6	IVIg, MP, MMF
7.9	LIP, HS	12.3	1.7	268					ABCT, IVIg

Abbreviations: ABCT, abatacept; AIHA, autoimmune haemolytic anaemia; CMV, cytomegalovirus; GCV, ganciclovir; HS, hepatosplenomegaly; ITP, immune-mediated thrombocytopenia; IvIg, intravenous immunoglobulin; LIP, lymphocytic interstitial pneumonitis; MMF, mycophenolate mofetil; MP, methylprednisolone; OM, otitis media; RTX, rituximab; T1D, type 1 diabetes; VCZ, voriconazole.
